# A Hybrid alldifferent-Tabu Search Algorithm for Solving Sudoku Puzzles

**DOI:** 10.1155/2015/286354

**Published:** 2015-05-20

**Authors:** Ricardo Soto, Broderick Crawford, Cristian Galleguillos, Fernando Paredes, Enrique Norero

**Affiliations:** ^1^Pontificia Universidad Católica de Valparaíso, 2362807 Valparaíso, Chile; ^2^Universidad Autónoma de Chile, 7500138 Santiago, Chile; ^3^Universidad Cientifica del Sur, Lima 18 Lima, Peru; ^4^Universidad Central de Chile, 8370178 Santiago, Chile; ^5^Universidad San Sebastián, 8420524 Santiago, Chile; ^6^Escuela de Ingeniería Industrial, Universidad Diego Portales, 8370109 Santiago, Chile; ^7^Facultad de Ingeniería, Universidad Santo Tomás, 2561694 Viña del Mar, Chile

## Abstract

The Sudoku problem is a well-known logic-based puzzle of combinatorial number-placement. It consists in filling a *n* ^2^ × *n* ^2^ grid, composed of *n* columns, *n* rows, and *n* subgrids, each one containing distinct integers from 1 to *n* ^2^. Such a puzzle belongs to the NP-complete collection of problems, to which there exist diverse exact and approximate methods able to solve it. In this paper, we propose a new hybrid algorithm that smartly combines a classic tabu search procedure with the alldifferent global constraint from the constraint programming world. The alldifferent constraint is known to be efficient for domain filtering in the presence of constraints that must be pairwise different, which are exactly the kind of constraints that Sudokus own. This ability clearly alleviates the work of the tabu search, resulting in a faster and more robust approach for solving Sudokus. We illustrate interesting experimental results where our proposed algorithm outperforms the best results previously reported by hybrids and approximate methods.

## 1. Introduction

**Figure 1:**
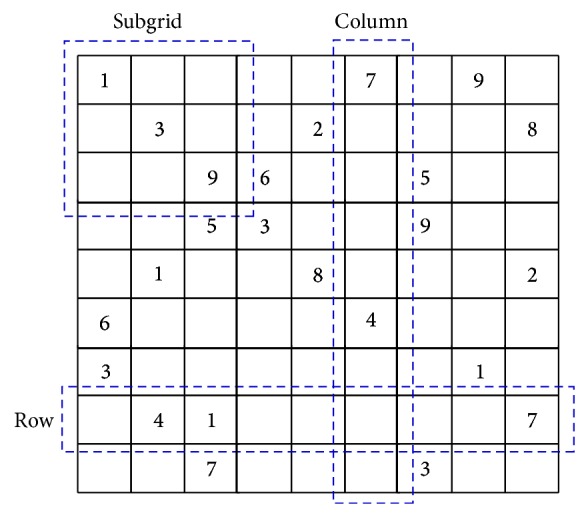
Sudoku puzzle instance: AI Escargot.

The Sudoku puzzle is a combinatorial problem consisting of assigning *n* ^2^ digits, from 1 to *n* ^2^, in each cell matrix of size *n* ^2^ × *n* ^2^. The matrix is composed of *n* ^2^ rows, *n* ^2^ columns, and *n* ^2^ subgrids of size *n* × *n*, as shown in Figure 1.

The problem has a set of simple rules; in each region every digit must be assigned only one time, and hence all digits must be assigned in each cell of each region. Any digit will be repeated *n* ^2^ times scattered across the grid but not repeated in same rows, columns, and subgrids. The common size of Sudoku is *n* = 3; thus the puzzle is defined as a 9 × 9 matrix with nine 3 × 3 subgrids. Each Sudoku instance starts with some values, called the* givens* and the difficulty of the puzzle depends on the positioning of those givens along the matrix. An instance has a unique solution if it contains at least 17 givens [1].

During the last years, Sudokus have appeared as interesting problems to test constraint satisfaction and optimization algorithms because of their NP-completeness and different modeling capabilities. In this context, several approaches from different domains have been proposed. For instance, exact methods such as constraint programming [2, 3] and SAT [4] are in general efficient techniques to solve Sudokus. On the approximate methods domain, metaheuristics have proven to be efficient as well [5, 6]. Some hybrids combining exact and approximate methods have also been reported [7, 8], as well as techniques such as Sinkhorn balancing [9], rewriting rules [10], and entropy minimization [11].

In this work, we introduce a new hybrid that smartly integrates a global constraint, namely, the alldifferent constraint, in a classic tabu search procedure. The alldifferent constraint comes from the constraint programming world and has specially been designed for the efficient domain reduction of variables involved in constraints that must be pairwise different. This global constraint works perfectly for Sudokus since all the puzzle constraints can be expressed as a pairwise comparison. We implement the alldifferent constraint following Puget's approach [12], which identifies Hall intervals [13] and then filters the domains. This allows one to efficiently propagate the constraints, considerably reducing the search space and alleviating the work of the tabu search. As a consequence, the search process is accelerated, while the quality of solutions is maintained. We illustrate interesting experimental results where our proposed algorithm outperforms the best results reported in the literature.

This paper is organized as follows. In Section 2, we describe the previous work.  Section 3 presents the classic tabu search. The alldifferent constraint is presented in Section 4. The proposed algorithm is presented in Section 5, followed by the corresponding experimental results. Finally, we conclude and give some directions for future work.

## 2. Related Work

In this paper, we concentrate on incomplete search methods, specially on solving hard instances of the puzzle. Within this scenario, different approaches have been suggested, mainly based on metaheuristics. For instance, in [14], the Sudoku puzzles are modeled as a combinatorial optimization problem where the objective function is the minimization of the incorrectly placed numbers on the board. The previous model is solved by using simulated annealing, but the approach is mostly focused on producing valid Sudokus than on the performance of the resolution. In [15], where a particle swarm optimizer (PSO) for solving Sudokus is presented, the goal of authors was to validate the use of geometric operators for PSO for complex combinatorial spaces. In [5], a classic genetic algorithm is tuned with similar geometric operators, particularly Hamming space crossovers and swap space crossovers, reporting good solutions for a hard Sudoku instance. In [16], another GA is presented improving the selection, crossover, and mutation operators. They achieve a better convergence rate and stability with respect to the classic GA. In [8], a hybrid combining AC3 and tabu search is reported, where the idea is to apply AC3 at each iteration of the metaheuristic in order to systematically attempt to reduce the variable domains. A similar approach using cuckoo search is presented in [7], but the AC3 is only employed as a preprocessing phase.

In [17], the alldifferent constraint is used to reduce variable domains by overlapping the 27 Sudoku constraints. The approach succeeds for easy instances and some other ones, but in more complex instances the solution is reached with a complete search solver. In Section 6, a comparison of the proposed algorithm with respect to the best results reached by hybrids and approximate methods is given.

## 3. Tabu Search

Tabu search (TS), introduced by Glover [18, 19], is a metaheuristic mainly concerned with combinatorial optimization problems. TS has successfully been employed for working on different kind of real-life problems as well as problems from operation research and computer science, such as the traveling salesman problem, the knapsack problem, and the timetabling problem.

TS is based on local search over single solutions, employing a given solution *S* as a start point. Then, this starting solution will be improved across small changes, being those solutions called “neighbors” of *S*, iteratively until some stopping criterion has been reached. The local search moves from neighbor to neighbor as long as possible according to a minimization/maximization of a defined objective function. Normally there exist problems with some moves so that *S* may be trapped in local optimum where the local search cannot find any further neighborhood solution.

To tackle the previous problem, TS makes use of a memory structure named tabu list, which is a feature that distinguishes it from other incomplete methods. The aim of the tabu list is

- to evade poor-scoring areas;
- to dodge unpromising areas and return to previously reached ones.

Hence, in the tabu list some data is kept of the recently visited solutions, with the aim of avoiding them if these are bad solutions, and so improving the efficiency of the search process. The tabu list is considered the most important feature of TS.

Algorithm 1 describes the classic procedure of TS. As input, the algorithm receives a primary solution that includes the givens values and an empty cell in the other positions, and as output it returns the best solution scored. At line 3, a* while* statement manages the iterations of the process until the defined stop criteria is reached. For instance, the stop condition is a maximum iteration limit or a threshold on the evaluation function. In this implementation, we use as evaluation function the minimization of remaining values to complete the puzzle. At line 7, new potential solutions are created by a neighboring procedure, these solutions are added to the candidate list exclusively if they do not include new solution elements on the tabu list. Then, a promising best candidate is selected on condition which is the best quality solution according to the evaluation by the cost function. At line 11, the cost evaluation of the chosen candidate is compared. If it improves the best solution (*S*_*best*_) cost, the differences of those are added to the tabu list and the *S*_*candidate*_ becomes the new *S*_*best*_.

Finally, some features are expired in the tabu list, and generally in the same order they were included, permitting in next iterations to add solutions to the candidate list which contains the expired features.

## 4. CP Overview and the alldifferent Constraint

Constraint programming (CP) is a paradigm for solving combinatorial search and optimization problems mainly from domains such as scheduling, planning, and vehicle routing. In CP, a problem is modeled by relating all involved variables of the problem in constraints terms, and a constraint solver is employed to solve it.

CP consists in two identifiable stages:

- Modeling: stating constraints involving the problem variables;
- Solving: finding a solution satisfying all the constraints.

Hence, all problems are represented in terms of decision variables and constraints, and the aim of the constraint solver is to find an assignment to all the variables that satisfies all the constraints.

### 4.1. Basic Concepts

A Constraint Satisfaction Problem (CSP) is defined by:

- A finite set of variables, *X* = *x*_1_, *x*_2_,…, *x*_*n*_.
- A domain for each variable, *D*(*X*) = *D*(*x*_1_), *D*(*x*_2_),…, *D*(*x*_*n*_), also noted as *d*_1_, *d*_2_,…, *d*_*n*_, where *d*_*i*_ is the domain of *x*_*i*_.
- A finite set of constraints *C*(*X*), where *c*(*x*_1_,…, *x*_*n*_) denotes a constraint involving variables *x*_1_,…, *x*_*n*_.

A CSP is denoted by the tuple *P*〈*X*, *D*, *C*〉. A CSP has solution only if every constraint in *C* is satisfied, and it is called a consistent CSP; further if no solution exists, it is an inconsistent CSP. Algorithms based on backtracking such as the forward checking are in general employed to solve CSPs. [20].

### 4.2. alldifferent Constraint

The alldifferent constraint commonly appears in problems which are based on permutations or when disjoint paths need to cover a directed graph [21–23], among other problems that involve constraints of pairwise difference. The main ability of this constraint is that it exploits the global information of the problem constraint, instead of handling each pairwise constraint independently. Exploiting the whole information leads to a more efficient domain filtering as explained in [24]. In the following, we provide some necessary definitions.

**Definition 1 .**

A *k*-ary constraint connecting variables in *X* with domains *D*(*X*) is defined as a subset of the cartesian product ∀*d* ∈ *D*(*X*) and it is intended as the set of allowed *k*-tuples for these *k* variables.

A constraint that involves one variable (e.g., 1-ary: *x*_1_ = 8) is called unary constraint and a binary one (e.g., 2-ary: *x*_1_ ≠ *x*_2_) involves 2 variables, and so on. In general, a *k*-ary constraint has a scope of size *k*. A conjunction of several simpler constraints is called a global constraint providing a more simple model for a problem; one of these constraints is the well-known alldifferent constraint.

The alldifferent constraint is a constraint of difference between all variables involved in the variable relation and specified that the value assigned to the variables must be pairwise different.

**Definition 2 .**

Let *x*_1_, *x*_2_,…, *x*_*n*_ be variables with respective finite domains *D*(*x*_1_), *D*(*x*_2_),…, *D*(*x*_*n*_), then

(1) alldifferentx1,…,xn=d1,…,dn ∣ ∀idi∈Dxi,  ∀i≠jdi≠dj.

Since the introduction of the alldifferent constraint [25], several filtering algorithms have been developed [24], depending on the desired degree of local consistency from “weaker” local consistency with low degree of filtering but short-time to “stronger” with an efficient filtering in a longer runtime. In this work, we employ the alldifferent constraint based on bounds consistency [12] and Hall's marriage theorem [13]. This implementation provides stronger propagation behavior, checking for exhaustion of all subranges of possible values [24].

**Definition 3 (bounds consistency).**

Let *c* be a constraint *c*(*x*_1_, *x*_2_,…, *x*_*n*_) with *n* > 1; a CSP is bounds consistent if for all variables and each value *d*_*i*_ from its domain, *d*_*i*_ ∈ {min*D*(*x*_*i*_), max*D*(*x*_*i*_)}, there exist values *d*_*j*_ ∈ [min*D*(*x*_*j*_), max*D*(*x*_*j*_)] for all *j* ∈ {1,…, *n*} − *i* such that (*d*_1_, *d*_2_,…, *d*_*n*_) ∈ *C*. min*D* and max*D* represent the minimum and maximum value, respectively, from the domain *D*.

**Definition 4 (Hall's theorem).**

Let *X* be a set of variables and *D* the corresponding finite variable domains. Suppose *G* is a bipartite graph with bipartition (*X*, *D*). There exists a matching that covers *X* if and only if for every subset *I*⊆*D*, |*D*(*I*)| ≤ |*I*| is fulfilled. Then *I* is called a Hall interval if |*I*| = |*K*_*I*_| with *K*_*I*_ = {*x*_*i*_∣*D*_*i*_⊆*I*}.

**Theorem 5 .**

The constraint $alldifferent(x_{1},\ldots ,x_{n})$ is bounds consistent if and only if {*d*_*i*_ ≥ 1∣∀ *d*_*i*_ ∈ *D*} and

- for each interval *I*: |*I* | ≥|*K*_*I*_|,
- for each Hall interval *I* : {min*D*_*i*_, max*D*_*i*_}∩*I* = *∅*; ∀*x*_*i*_ ∉ *K*_*I*_.

**Proof**

We proceed by induction, observing that the case |*I*| = 1 obviously holds, because all domains are greater than 1. Let I be a Hall interval and *x*_*i*_ ∉ *K*_*I*_. If alldifferent(*x*_1_, *x*_2_,…, *x*_*n*_) is bounds consistent, it has a solution when *x*_*i*_ = min*D*_*i*_, by Definition 3.

**Example 6 .**

**Figure 2:**
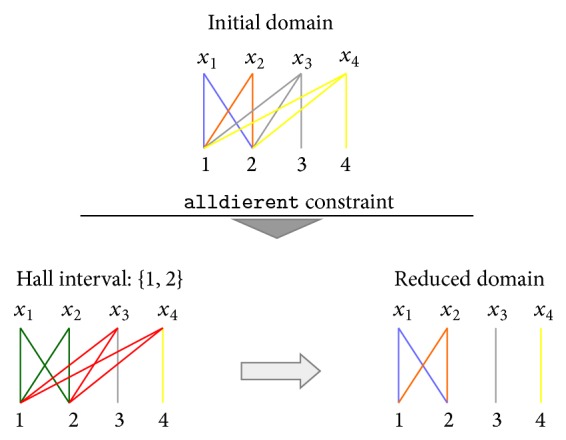
Example of alldifferent constraint through Hall's theorem approach.

Consider the following CSP which is represented in Figure 2:

(2) x11,2,x2∈1,2,x3∈1,2,3,x4∈1,2,4alldifferentx1,x2,x3,x4.

In order to make bounds consistency of the CSP, the inconsistent values must be removed. The algorithm founds Hall intervals, in this case when the interval is set to {1,2}. Since |*I* | = 2 and to satisfy the theorem, *I* : {min*D*_*i*_, max*D*_*i*_}∩*I* = *∅*, the interval must be removed from *x*_3_ and *x*_4_ domains.

The new domains for all variables will be

(3) x11,2,x2∈1,2,x3∈3,x4∈4.

In summary, there exists a solution to an alldifferent constraint if and only if for each subset of variables, the union of their domains holds the adequate values to match every one of them with a distinct value. In the previous example, when the Hall interval is set to *I* = {1,2}, being represented by green and red lines, we note that the values from *x*_1_ and *x*_2_ domains cannot be assigned to any other variable. Hence, the values of the interval *I* are removed from *x*_3_ and *x*_4_ variables. The reduced domains are only sets with feasible values with respect to all constraints.

**Example 7 .**

**Figure 3:**
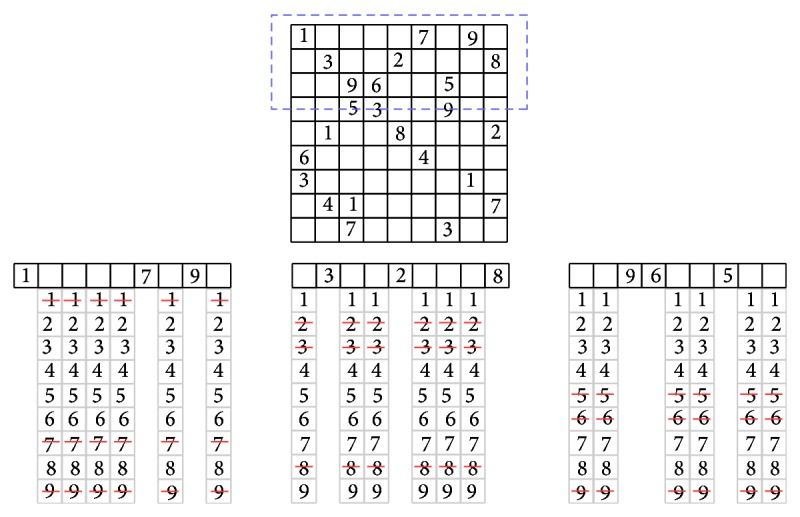
alldifferent constraint applied to the rows of AI Escargot instance. Only the first three rows are shown.

We illustrate with another example applying the alldifferent constraint on the most difficult Sudoku instance, and it is called AI Escargot ([28]). Each Sudoku instance is composed of three types of constraints: row, columns, and subgrid. We begin by enforcing the alldifferent constraint on the rows of the Sudoku puzzle. We have used just 3 constraints corresponding to the first three Sudoku rows (enclosed with dashed lines in Figure 3) instead of the nine ones in order to simplify the illustration, but the filtering technique is applied to all constraints.

After applying the alldifferent constraint from the first to third rows, the domains are only reduced but no value is discovered due the difficulty of the instance. The values deleted from the reduced domains did not satisfy the constraint; they have been already taken for another cell on the same row.

**Figure 4:**
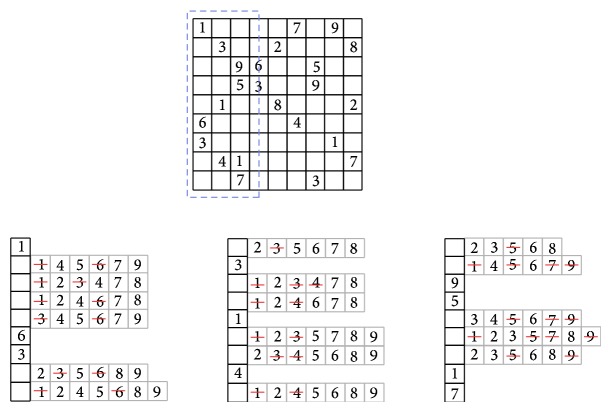
alldifferent constraint applied to the columns of the AI Escargot instance, using the reduced domains of variables in common with the previous reduced ones. Only the first three columns are shown.

In Figure 4, the alldifferent constraint is applied to columns of the puzzle. Only the first three columns are shown.

**Figure 5:**
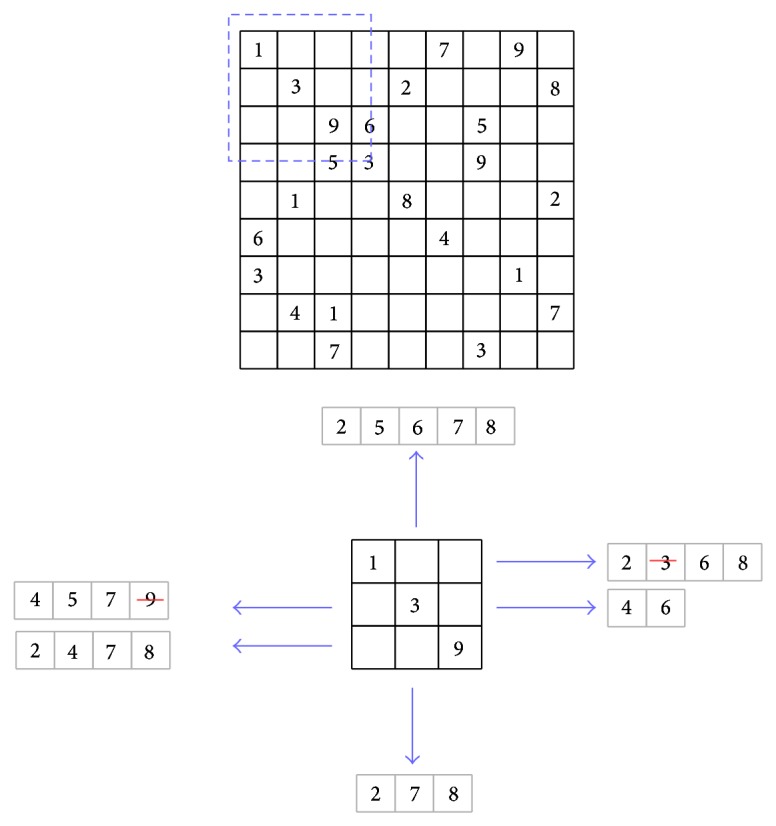
alldifferent constraint applied to the subgrids of AI Escargot instance, using the reduced domains of variables in common with the previous reduced ones. Only the first subgrid is shown.

In Figure 5, the alldifferent constraint is applied to the subgrids of the puzzle; the reduced domains by the previous domain filterings are used. At this point, the overall domain size of the subgrid has been reduced from 57 elements to 20, being more than 64% of domain reduction, significantly decreasing the amount of possible assignments.

## 5. Proposed Algorithm

The main idea of the proposed algorithm (Algorithm 2) is to employ the alldifferent constraint so as to filter the concerned variable domains as a preprocessing phase (line 1) and at every iteration of the Tabu search (line 9). The alldifferent constraint is applied iteratively over all structures of the grid (rows, columns, and subgrids). In the preprocessing phase and within each iteration some values are deleted from unfeasible regions, easing the work of the search process of the metaheuristic.

At line 6, we have limited the search neighboring procedure to assignments from the filtered domain. The randomization is still used, but just for randomizing the value selection of filtered values.

As stop condition (line 3), we use the full coverage of the grid, and it means that solution is found and a maximum of iterations which has been fixed to 10,000.

As output, the procedure returns *S*_*best*_, which is the outstanding solution achieved by the algorithm.

## 6. Experimental Results

In this section, we present a performance evaluation of the proposed algorithm to solve Sudokus. The tested benchmarks are classified in diverse kinds of complexity, including the AI Escargot which is considered the most difficult instance [28]. A useful difficulty classification including easy, medium, and hard Sudoku instances has been proposed in [26]. Here, we extend this classification in order to incorporate additional instances reported in the literature [29, 30]. The reclassification is depicted in Table 1. All difficulty classification groups have 3 instances (a, b, and c) per subgroup, except for the AI Escargot which is a single instance. All instances have unique solution.

Firstly, we have compared the filtering technique used in previous work [7, 8]. As mentioned before, this phase is very important and useful to reduce the search space, consequently facilitating the metaheuristic work. The filtering technique employed was arc consistency-3 (AC-3) [31]. AC-3 examines the arcs between pairs of variables and removes those values from the domains which are not consistent with the involved constraints. If a domain of a variable changes, the involved arcs of the variable, which its domain has been recently reduced, are examined again to check the arc consistency of the reduced domain.

Table 2 illustrates the percentage of domain reduction for each Sudoku instance. The results exhibit the fact that the alldifferent constraint outperforms the AC-3 algorithm in terms of filtering capabilities. This is produced due to the ability of the alldifferent constraint to employ the global information of the pairwise constraints instead of handling the constraints independently as the AC3 does. Let us note that the ability to infer a greater number of elements which do not belong to the domain of the problem solution depends only on the problem constraints. The alldifferent constraint and the characteristics of the problem, |*X*| = *n*, enable the use of Hall's theorem to infer the reduction of domains until each domain variable has one element (in the best case) by eliminating the elements of the domains in which they never be part of any (the only) solution.

Table 3 depicts the required runs to successfully solve 30 times each Sudoku instance considering 10,000 iterations as limit. The symbol ↑50 indicates that more than 50 runs are needed to successfully solve 30 times the instance. We contrast the proposed approach with the best performing algorithms reported in the literature (AC3-TS [8], AC3-CS [7], and GA [26, 29]). The results for easy instances show no relevant differences. However, for medium, SD, and hard instances, the performance of the proposed alldiff-TS is greatly better.

**Figure 6:**
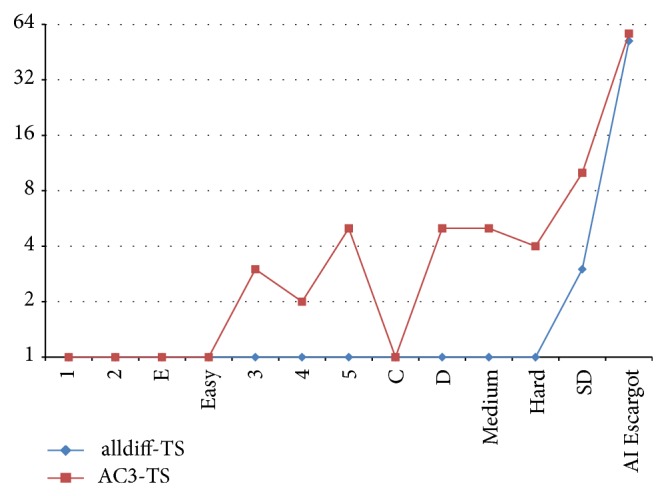
Comparing minimum needed iterations for Sudoku solving.

**Figure 7:**
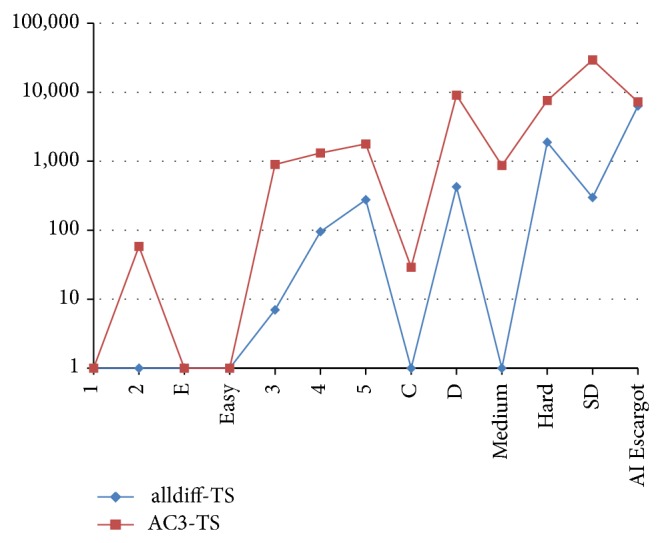
Comparing maximum needed iterations for Sudoku solving.

**Figure 8:**
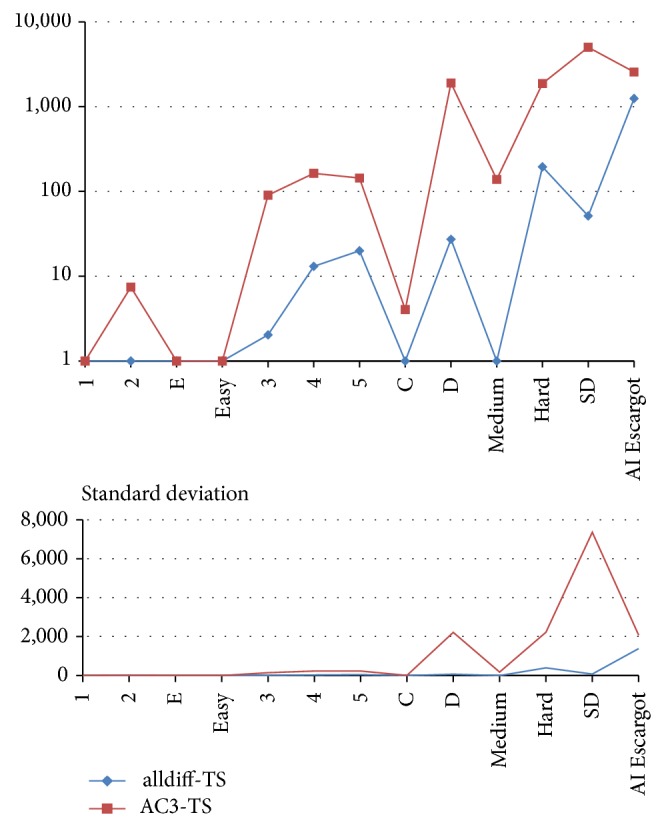
Comparing average needed iterations for Sudoku solving and their standard deviations.

Table 4 contrasts the proposed approach with AC3-TS, which is the best performing one from previously reported approaches. We compare the minimum, average, and maximum iterations needed to successfully solve each Sudoku instance. We consider 30 runs for each instance. The results exhibit the fact that alldiff-TS achieves the constraint satisfaction of all tested instances requiring considerable less iterations than AC3-TS (A graphical comparison can be seen in Figures 6, 7, and 8). Let us remark that TS has a high participation in the search process and the work is not only done by the filtering technique.

## 7. Conclusion and Future Work

In this paper, we have presented a new hybrid that integrates the powerful alldifferent constraint into a classic tabu search algorithm. The alldifferent constraint is employed to efficiently delete the values from domains that do not conduct to any feasible solution. The role of this filter is to act prior to the TS procedure but also in the search cycle, which permits progressive filtering of the best solutions. This allows us to relieve the work of the metaheuristic in order to achieve faster solving processes. We have carried out a set of experiments in order to contrast our approach with the best performing approximate methods and hybrids reported in the literature. We have considered different complexity instance Sudokus, including the AI Escargot which is considered the most difficult one. The result has exhibited encouraging results, where the proposed approach noticeably outperforms the previous algorithms reported in the literature.

A clear direction for future work is to study the integration of the alldifferent constraint on additional metaheuristics and to contrast performance. Particularly, the addition of global constraints on a cuckoo search [32] algorithm would be a promising hybrid, given the fact that CS algorithm has exhibited great performance and has already been combined with filtering techniques. Another interesting research direction will be the study of variations of the classic alldifferent constraint working in conjunction with approximate methods. An example is the symm  alldifferent constraint [24], which will be useful in the resolution of the well-known round-robin tournament.

## Figures and Tables

**Algorithm 1:**
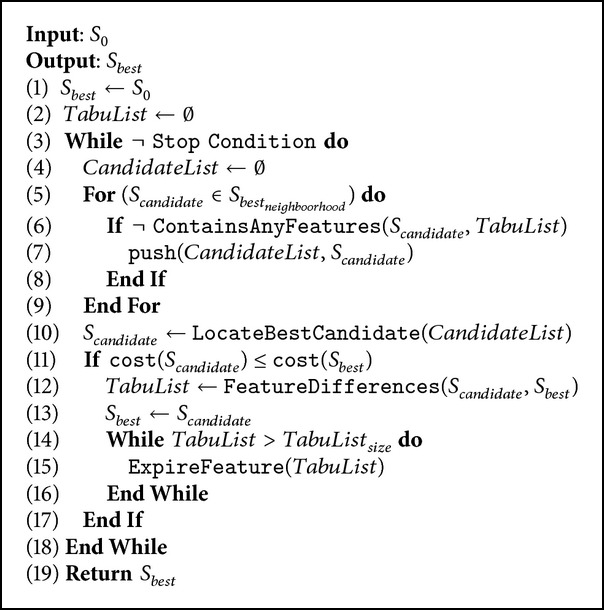
Tabu Search.

**Algorithm 2:**
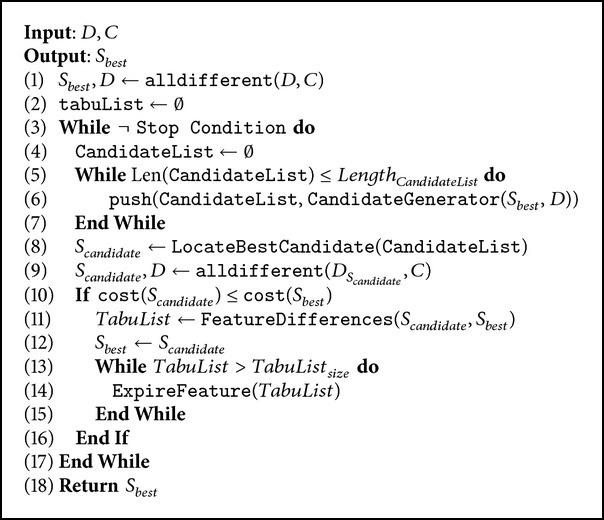
Hybrid a lldifferent-Tabu search.

**Table 1:** Difficulty of tested instances.

Authors	Difficulty groups			
	Easy	Medium	Difficult	Most difficult
[26]	1, 2	3, 4, 5	—	—
	E	C, D	SD	—
[27]	Easy	Medium	Hard	—
[28]	—	—	—	AI Escargot

**Table 2:** Comparison of effectiveness by filtering techniques.

Difficulty group	Difficulty subgroup	% Domain reduction			% Domain reduction		
		a lldifferent			AC-3		
		*a*	*b*	*c*	*a*	*b*	*c*
Easy	1	100%	100%	100%	100%	100%	100%
	2	100%	100%	100%	79%	100%	100%
	E	100%	100%	100%	100%	100%	100%
	Easy	100%	100%	100%	100%	100%	100%

Medium	3	100%	100%	89%	92%	74%	82%
	4	86%	100%	82%	75%	75%	76%
	5	87%	100%	80%	73%	74%	79%
	C	100%	100%	100%	88%	100%	100%
	D	75%	100%	100%	68%	73%	77%
	Medium	100%	100%	100%	81%	78%	76%

Difficult	SD	80%	80%	84%	69%	68%	68%
	Hard	68%	87%	100%	66%	74%	68%

Most difficult	AI Escargot	71%			70%		

**Table 3:** Solving Sudokus considering 10,000 iterations as maximum.

Difficulty group	Difficulty subgroup	Tries			Tries			Tries			Tries		
		a lldiff-TS			AC3-TS			AC3-CS			GA		
		*a*	*b*	*c*	*a*	*b*	*c*	*a*	*b*	*c*	*a*	*b*	*c*
Easy	1	30	30	30	30	30	30	30	30	30	—	—	—
	2	30	30	30	30	30	30	↑ 50	30	30	—	—	—
	E	30	30	30	30	30	30	30	30	30	—	—	—
	Easy	30	30	30	30	30	30	30	30	30	30	30	30

Medium	3	30	30	30	30	30	30	48	↑ 50	↑ 50	—	—	—
	4	30	30	30	30	30	30	↑ 50	↑ 50	↑ 50	—	—	—
	5	30	30	30	30	30	30	↑ 50	↑ 50	↑ 50	—	—	—
	C	30	30	30	30	30	30	—	—	—	—	—	—
	D	30	30	30	41	30	30	—	—	—	—	—	—
	Medium	30	30	30	30	30	30	↑ 50	↑ 50	↑ 50	22	—	—

Difficult	SD	30	30	30	30	30	↑ 50	—	—	—	—	—	—
	Hard	30	30	30	30	30	46	↑ 50	↑ 50	—	2	—	—

Most difficult	AI Escargot	30			30			—			—		

**Table 4:** Iterations needed (minimum, average, maximum, and standard deviation) considering 30 runs.

Difficulty group	Difficulty subgroup	Iterations							
		a lldiff-TS				AC3-TS			
		Minimum	Average	Maximum	*σ*	Minimum	Average	Maximum	*σ*
Easy	1	1	1	1	0	1	1	1	0
	2	1	1	1	0	1	7.4	58	11.7
	E	1	1	1	0	1	1	1	0
	Easy	1	1	1	0	1	1	1	0

Medium	3	1	2.0	7	1.6	3	90.2	897	140.5
	4	1	13.1	95	23.5	2	163.3	1,318	226.3
	5	1	20	276	45.0	5	143.9	1,778	230.4
	C	1	1	1	0	1	4.0	29	6.0
	D	1	27.2	424	64.5	5	1,897.6	9,088	2,214.3
	Medium	1	1	1	0	5	138.8	865	173.9

Difficult	SD	3	51.5	298	68.8	10	5,005.6	29,329	7,358.1
	Hard	1	195.0	1881	397.0	4	1,872.4	7,590	2,217.6

Most difficult	AI Escargot	52	1,248.3	6,328	1,378.6	57	2,566.7	7,224	2,076.6
